# Comparative analysis of machine learning algorithms for multi-syndrome classification of neurodegenerative syndromes

**DOI:** 10.1186/s13195-022-00983-z

**Published:** 2022-05-03

**Authors:** Leonie Lampe, Sebastian Niehaus, Hans-Jürgen Huppertz, Alberto Merola, Janis Reinelt, Karsten Mueller, Sarah Anderl-Straub, Klaus Fassbender, Klaus Fliessbach, Holger Jahn, Johannes Kornhuber, Martin Lauer, Johannes Prudlo, Anja Schneider, Matthis Synofzik, Adrian Danek, Janine Diehl-Schmid, Markus Otto, FTLD-Consortium Germany, Arno Villringer, Karl Egger, Elke Hattingen, Rüdiger Hilker-Roggendorf, Alfons Schnitzler, Martin Südmeyer, Wolfgang Oertel, German Atypical Parkinson Consortium Study Group, Jan Kassubek, Günter Höglinger, Matthias L. Schroeter

**Affiliations:** 1 AICURA Medical GmbH, Berlin, Germany; 2 Clinic for Cognitive Neurology, University Clinic Leipzig, Leipzig, Germany; 3 Max Planck Institute for Human Cognitive and Brain Sciences, Leipzig, Germany; 4 Carl Gustav Carus Faculty of Medicine, Institute for Medical Informatics and Biometry, TU Dresden, Dresden, Germany; 5 Swiss Epilepsy Clinic, Klinik Lengg, Zurich, Switzerland; 6 Department of Neurology, University of Ulm, Ulm, Germany; 7 Department of Neurology, Saarland University, Homburg, Germany; 8 Clinic for Neurodegenerative Diseases and Geriatric Psychiatry, German Center for Neurodegenerative Diseases (DZNE), University of Bonn, Bonn, Germany; 9 Clinic for Psychiatry and Psychotherapy, University Hospital Hamburg-Eppendorf, Hamburg, Germany; 10 Department of Psychiatry and Psychotherapy, Friedrich-Alexander-University of Erlangen-Nuremberg, Erlangen, Germany; 11 Department of Psychiatry and Psychotherapy, University Wuerzburg, Würzburg, Germany; 12 Department of Neurology, DZNE, University of Rostock, Rostock, Germany; 13 Department of Neurodegenerative Diseases, Centre for Neurology & Hertie-Institute for Clinical Brain Research, University of Tuebingen, Tübingen, Germany; 14 DZNE, Tübingen, Germany; 15 Department of Neurology, Ludwig-Maximilians-Universität München, Munich, Germany; 16 Department of Psychiatry and Psychotherapy, Technical University of Munich, Munich, Germany; 17 Department of Neuroradiology, University Hospital of Freiburg, Freiburg, Germany; 18 Department of Neuroradiology, University Hospital of Frankfurt, Frankfurt, Germany; 19 Department of Neurology, Klinikum Vest, Recklinghausen, Germany; 20 Institute of Clinical Neurosciences and Medical Psychology, Heinrich Heine University of Düsseldorf, Düsseldorf, Germany; 21 Department of Neurology, Ernst von Bergmann Klinikum, Potsdam, Germany; 22 Department of Neurology, Philips-University Marburg, Marburg, Germany; 23 Department of Neurology, Technical University of Munich, Munich, Germany; 24 German Center for Neurodegenerative Diseases, Munich, Germany

**Keywords:** Multi-syndrome classification, Neurodegenerative syndromes, Deep neural network, Comparative analysis, Support vector machine, Random forest, Gradient boosting

## Abstract

**Importance:**

The entry of artificial intelligence into medicine is pending. Several methods have been used for the predictions of structured neuroimaging data, yet nobody compared them in this context.

**Objective:**

Multi-class prediction is key for building computational aid systems for differential diagnosis. We compared support vector machine, random forest, gradient boosting, and deep feed-forward neural networks for the classification of different neurodegenerative syndromes based on structural magnetic resonance imaging.

**Design, setting, and participants:**

Atlas-based volumetry was performed on multi-centric T1-weighted MRI data from 940 subjects, i.e., 124 healthy controls and 816 patients with ten different neurodegenerative diseases, leading to a multi-diagnostic multi-class classification task with eleven different classes.

**Interventions:**

N.A.

**Main outcomes and measures:**

Cohen’s kappa, accuracy, and F1-score to assess model performance.

**Results:**

Overall, the neural network produced both the best performance measures and the most robust results. The smaller classes however were better classified by either the ensemble learning methods or the support vector machine, while performance measures for small classes were comparatively low, as expected. Diseases with regionally specific and pronounced atrophy patterns were generally better classified than diseases with widespread and rather weak atrophy.

**Conclusions and relevance:**

Our study furthermore underlines the necessity of larger data sets but also calls for a careful consideration of different machine learning methods that can handle the type of data and the classification task best.

## Key points

Question: This study compares the different machine learning methods for predicting several neurodegenerative syndromes.

Findings: The comparison of support vector machine, random forest, gradient boosting, and deep feed-forward neural networks yielded the neural networks to be the best for the classification of different neurodegenerative syndromes based on pre-structured volume measures.

Meaning: Even with pre-structured data, deep neural networks are most promising.

## Introduction

In light of the demographic shift and the pending shortage of resources in healthcare systems across the globe, computer-aided methods are to shoulder some of the challenges. Supportive technology will find its way into the clinic to assist physicians in finding the correct diagnosis [1]. The implementation of artificial intelligence into clinical routine is happening already, and it is a matter of time until medical decisions will rely on algorithms in conjunction with the experience of physicians.

In case of neurodegenerative syndromes, brain imaging can render important MRI-morphological biomarkers in the form of atrophy patterns. While some focal atrophy patterns are quite disease-specific [2–8] leading even to incorporation into diagnostic criteria [9–11], neuroimaging findings for other diseases might be less conclusive [12]. However, it requires highly trained and specialized neuroradiologists to correctly detect and interpret the signs—an expertise that is not available ubiquitously.

For analyzing the complex multivariate and nonlinear relationships in high dimensional data derived from MRI data, machine learning algorithms are superior to standard inferential statistics [13, 14]. For the classification of neurological and psychiatric diseases, support vector machines (SVM) based on imaging-derived data have been the most popular method [14]. SVMs have proven to be a suitable approach at least in binary differentiations of patients from healthy controls [13–15]. A few studies further used SVM to differentiate disease entities from each other—a more complex approach that simulates the process of differential diagnosis. In a previous study, we assessed the performance of SVM to differentiate two dementia syndromes from each other [16, 17]. In another study, SVM was used to classify various parkinsonian syndromes based on the results of volumetric MRI analysis [18]. While SVM produced satisfactory results, other methodological approaches were not assessed further.

In recent years, deep learning methods have become more and more popular for pattern recognition tasks such as the classification of image and text data, but also of structured data [19]. Deep learning methods process data on several levels. In this way, more and more abstract representations are generated up to the class as the most abstract form of representation [19]. Deep neural networks (DNNs) in particular have proven to be highly proficient in predicting diagnoses based on imaging data of the eye, skin, or lung [20–22] and will most likely become a key component of imaging diagnostics in the future. Hopefully, these advanced models will be able to capture more complex atrophy patterns in the human brain than SVM approaches and might assist radiologists with their assessment in the future.

Accordingly, we will compare these models for the classification of neurodegenerative syndromes based on atlas-based volume measures in a very large dataset including numerous diseases in this work. Besides DNN and SVM, we will apply two ensemble learning methods (i.e., random forest (RF) and gradient boosting (GB)) that have been thriving as proficient models in many classification challenges dealing with similar data [14, 23]. The preprocessing of the data into structured data via volumetry in the form of an atlas is useful for clinical purposes, because it normalizes data, reduces thereby inter-center variability, guarantees a complete anonymization of the data, and decreases computing time when training the models.

The syndromes considered in this study all belong to the neurodegenerative disease spectrum ranging from Alzheimer’s disease (AD), frontotemporal lobar degeneration with its subtypes behavioral variant frontotemporal dementia (bvFTD), and primary progressive aphasias (PPA) with the three subforms—semantic variant (svPPA), nonfluent-agrammatic variant (nfvPPA), and logopenic variant (lvPPA)—to atypical Parkinson syndromes such as corticobasal syndrome (CBS), progressive supranuclear palsy (PSP), and multiple system atrophy with cerebellar features (MSA-C), as well as MSA with predominant parkinsonism (MSA-P) and idiopathic Parkinson’s disease (PD).

This use case is exemplary for imaging-derived structural data and can be transferred to other use cases of the biomedical sciences. By including ten different neurodegenerative diseases beside a control cohort, our approach mirrors best the work of radiologists in clinical routine, i.e., firstly categorizing a brain scan as normal or abnormal and secondly defining the neurodegenerative entity in the differential diagnostic process. We hypothesize (i) that neurodegenerative diseases can be classified with reasonable accuracy from structural brain imaging data, in particular, if they are characterized by specific atrophy patterns, and (ii) that DNNs perform better than SVM.

## Methods

### Subjects and demographic characteristics

The study included multi-centric data from 940 subjects, i.e., 124 healthy controls and 816 patients from the German Research Consortium of Frontotemporal Lobar Degeneration (www.ftld.de) [24] and from the German Atypical Parkinson Consortium Study Group [18, 25]. The patient cohort consisted of 72 patients with AD, 146 patients with bvFTD, 26 patients with CBS, 30 patients with lvPPA, 21 patients with MSA-C, 60 patients with MSA-P, 58 patients with nfvPPA, 203 patients with PD, 154 patients with PSP, and 46 patients with svPPA.

**Fig. 1:**
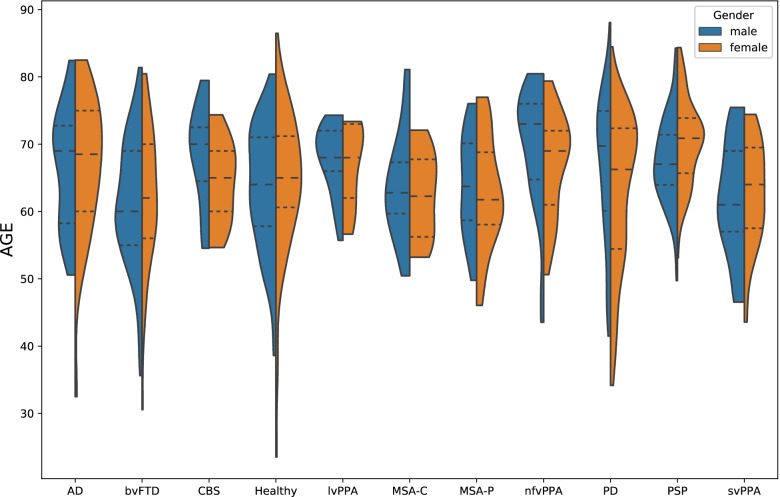
Violin plot of the age and gender distribution of the cohort sample. The dashed line indicates the mean, and the dotted line indicates the standard deviation. AD, Alzheimer’s disease; bvFTD, behavioral variant frontotemporal dementia; CBS, corticobasal syndrome; lvPPA, logopenic variant primary progressive aphasia; MSA-C, multiple system atrophy (cerebellar dysfunction subtype); MSA-P, multiple system atrophy (parkinsonian subtype); nfvPPA, nonfluent variant primary progressive aphasia; PD, Parkinson’s disease; PSP, progressive supranuclear palsy; svPPA, semantic variant primary progressive aphasia

Figure 1 and Table 1 provide an overview of the age and gender distribution of the study cohort. Age distribution was compared with the Kruskal-Wallis test and post hoc with a Wilcoxon rank-sum test between all pairs of samples (Bonferroni-corrected). Patients with AD were significantly older than patients with bvFTD (*p* < 0.05). Patients with PSP were significantly older than healthy controls (*p* < 0.001) and patients with MSA-C (*p* < 0.05), MSA-P (*p* < 0.001), PD (*p* < 0.05), bvFTD (*p* < 0.001), and svPPA (*p* < 0.001). Furthermore, patients with bvFTD were significantly younger than patients with nfvPPA (*p* < 0.001). Also, patients with svPPA were significantly younger than patients with nfvPPA (*p* < 0.05).

**Table 1:** Demographic characteristics for patients and healthy controls

	Number	Age (years)	Gender (female/male)
AD	72	66.67 (± 9.59)	39/33
bvFTD	146	61.68 (± 9.67)	53/93
CBS	26	65.96 (± 6.91)	15/11
lvPPA	30	67.33 (± 5.60)	13/17
MSA-C	21	63.05 (± 7.24)	11/10
MSA-P	60	63.29 (± 7.99)	38/22
nfvPPA	58	68.46 (± 8.32)	29/29
PD	203	64.08 (± 11.21)	135/68
PSP	154	69.03 (± 6.47)	82/72
svPPA	46	62.14 (± 8.31)	19/27
Healthy controls	124	63.71 (± 10.00)	60/64

Data are reported as mean ± standard deviation

*Abbreviations*: *AD* Alzheimer’s disease, *bvFTD* Behavioral variant frontotemporal dementia, *CBS* Corticobasal syndrome, *lvPPA* Logopenic variant primary progressive aphasia, *MSA-C* Multiple system atrophy (cerebellar dysfunction subtype), *MSA-P* Multiple system atrophy (parkinsonian subtype), *nfvPPA* Nonfluent variant primary progressive aphasia, *PD* Parkinson’s disease, *PSP* Progressive supranuclear palsy, *svPPA* Semantic variant primary progressive aphasia

Gender distribution was tested pairwise with the Fisher test (Bonferroni-corrected) post hoc if the chi-square test indicated significant differences (chi-square = 38.855, *p* < 0.001). The gender distribution significantly differed between patients with bvFTD and PD (*p* < 0.001), MSA-P (*p* < 0.05), and PSP (*p* < 0.05). Furthermore, there was a significant difference in gender distribution between patients with PD and svPPA (*p* < 0.05).

The study was conducted according to the Declaration of Helsinki. It was approved by the local ethics committees of all participating centers. Patients, participants, caregivers, or legal representatives gave written informed consent for the study.

### Imaging acquisition and analysis

Standardized structural MRI head scans were acquired multi-centrically at German university hospitals. Every subject obtained a T1-weighted three-dimensional (1-mm isovoxel resolution) magnetization-prepared rapid gradient echo (MPRAGE) head MRI brain scan [18, 24, 26]. The MPRAGE sequence was converted to ANALYZE 7.5 format, and the file names were pseudonymized before further processing. Whereas standardized operating procedures (SOPs) have been applied throughout the data acquisition including MRI in the German Research Consortium of Frontotemporal Lobar Degeneration, no sequence adjustment or homogenization between the centers was done in the German Atypical Parkinson Consortium Study. Instead, the MPRAGE sequence from the clinical routine at each center was used (for further information on MRI parameters, see papers and supplemental materials [4, 17, 18, 25, 27]). Atlas-based volumetric analysis of the MPRAGE sequence data was done using the LONI Probabilistic Brain Atlas (LPBA40) [28], and further masks were derived from this atlas. The atlas structures were used as an input vector for the model and represent the volume measures of the input data. A detailed description of all image processing steps and the 63 atlas structures included can be found in [18]. Before being used as predictive features, all volume results were corrected for intracranial volume (ICV).

### Training and evaluation of classifiers

**Fig. 2:**
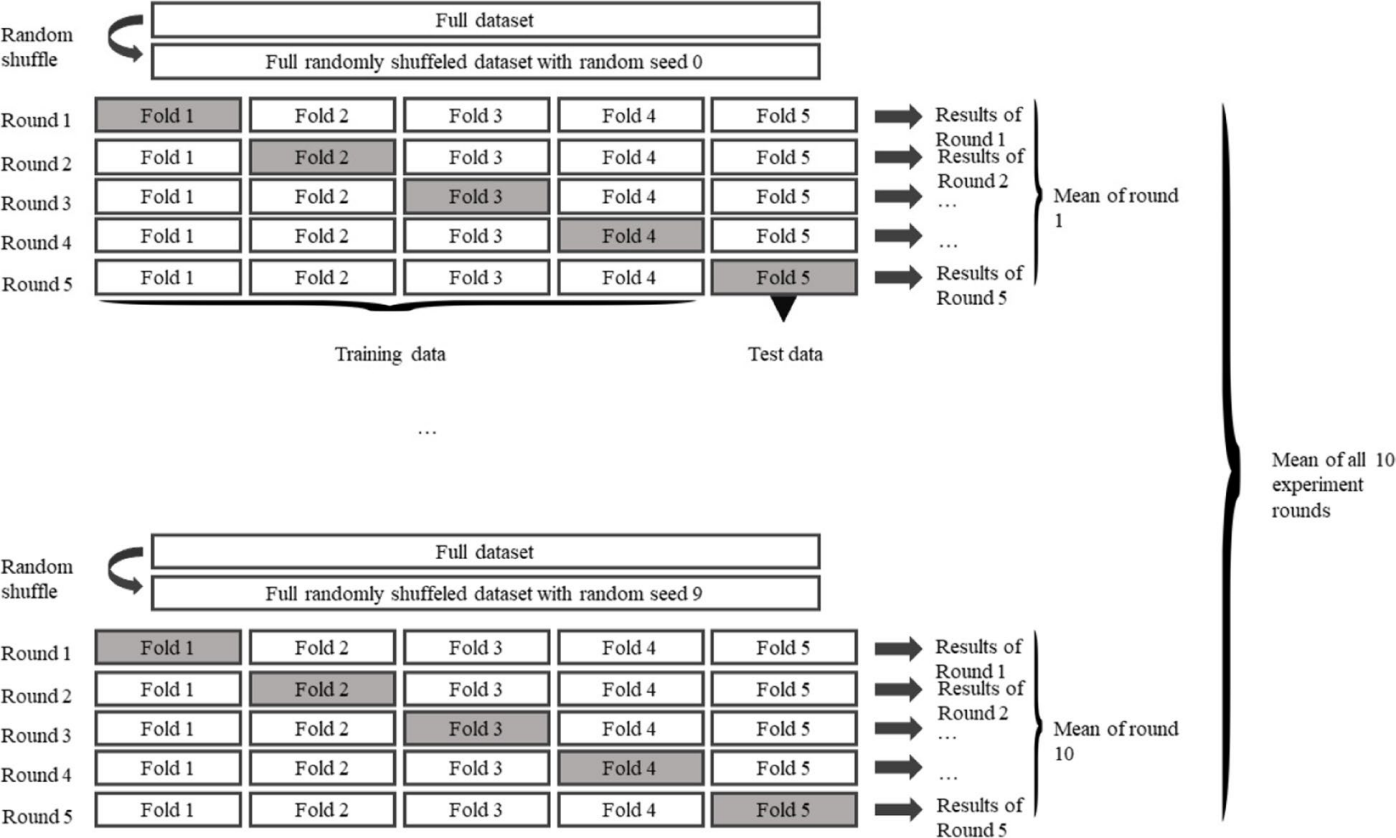
Design of the repeated 5-fold cross-validation. These experiments are repeated ten times, with the individual folds composed differently in each repetition. The random compositions are controlled by the random seeds

In order to reduce the bias of the existing sampling distribution, we used a 5-fold cross-validation with the full dataset (models were trained on 80% of the data (4-folds), 20% served for testing (1-fold)). The folds were selected randomly, and the experiments were repeated ten times. Thus, we trained and evaluated 50 models of each type (see Fig. 2). In each training iteration, we optimized the learning and hyperparameters of the RF model, the GB, and the SVM using Bayesian optimization. In contrast to a grid search or a random search, Bayesian optimization allows a sequential search and thus includes every previous search step. This leads to better optimization results [29]. During the optimization, overfitting was reduced by using a 5-fold cross-validation, where the training data was split into training and validation data. The optimization was run for 120 iterations.

The kernel applied in the SVM algorithm is critical for its way of functioning. Therefore, the learning and hyperparameters to be optimized also depend on the selected kernel. For this reason, the optimization of the SVM was done separately with linear, sigmoid, polynomial, and radial basis function kernels. Based on this comparison, we considered only SVM with a linear kernel. The linear kernel provided the best SVM performance, where *c* is optimized for avoiding misclassifications.

For the RF classifier, we optimized the maximum depth of the tree, the number of features to consider, the minimum number of samples required for a leaf node, and the minimum number of samples required to split an internal node. In case of GB optimization, we additionally optimized the learning rate.

We used a feed-forward DNN with 72 neurons in the input layer and 90 neurons in each of the two hidden layers. In order to prevent overfitting, we used a dropout rate [30] of 45% for the neurons of the hidden layers and early stopping [31]. For the weight update, we used Adam [32] and categorical cross-entropy, where the optimizer was initialized with *α* = 0.001, *β*_1_ = 0.9 =, *β*_2_ = 0.999, and *ϵ* = 10^−8^. The training was done batchwise with samples of 30 patients. More complex architectures have provided worse or similar classification results in the experiments.

The evaluation was done classwise considering the recall, the precision, and the F1-score. We dispense with a consideration of the overall recall, the overall precision, and the overall F-score, because of the included bias in the actual distribution, which limits the suitability of the F1-score for the model evaluation [33]. Therefore, this metric is not used for the overall model evaluation, but to show the distribution of predicted classes. To reduce this bias and evaluate the model performance, we use the Cohen’s kappa coefficient *κ* [34], which is defined as follows:

$$\kappa =\frac{y-{\hat{y}}}{1-{\hat{y}}}$$

where *y* denotes the ground truth syndrome classification and $\hat{y}$ the predicted syndrome. In addition, we show the accuracy, which allows a more humanly interpretable evaluation, but without considering the class imbalance. The accuracy is calculated for the total number of elements *n* in the test fold and has the following form:

$$\mathrm{accuracy}\left(y,{\hat{y}}\right)=\frac{1}{n}-1\left(y,{\hat{y}}\right)$$

To better understand the classification process of each model, we used a novel technique, namely “local interpretable model-agnostic explanations” (LIME), which explains the relationship between the components (here, brain regions) that are used for the classification and its predicted class (here, the syndrome) [35]. This method allowed the direct comparison of the decision process of all four models.

## Results

In the following, the model performance is presented with its classification results and the features, i.e., the brain regions, that have the highest value for the classification decision.

### Comparison of classification models

**Fig. 3:**
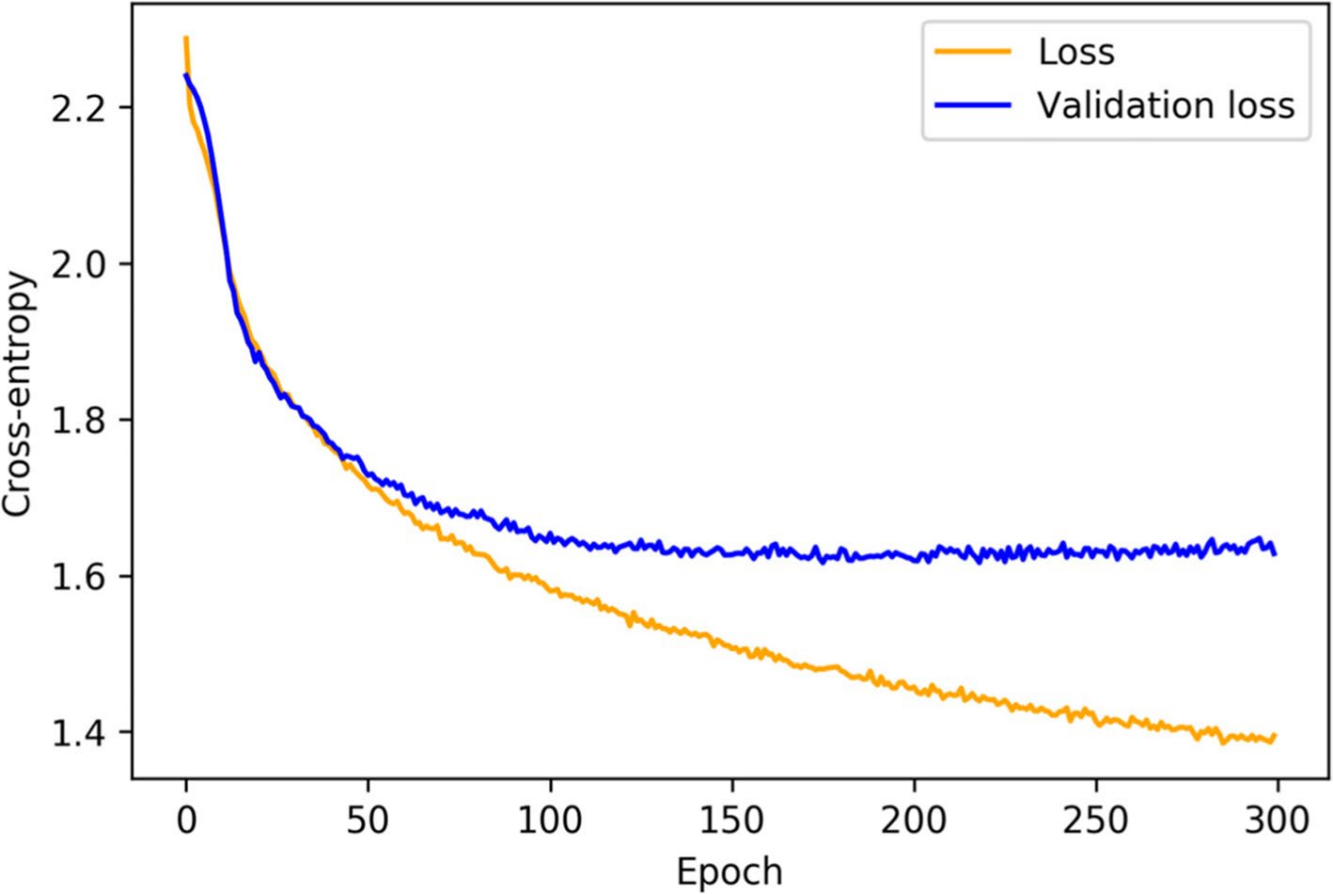
Averaged training loss and validation loss of the DNN. For the consideration of overfitting, the early stopping was dispensed with for this recording

The parameter settings for the tree-based methods (i.e., GB and RF) were found through the optimization processes after around 80 iterations, while the configuration for the SVM with linear kernel required only around 20 iterations. In the training of the DNN, the validation loss decreased until convergence, which was reached after about 100 epochs on average. The training is stopped, if the validation score was stable for 20 epochs. The convergence and the subsequent overfitting on the training data are shown in Fig. 3.

Model-wise performance measures can be found in Table 1. Among the models evaluated in this study, the DNN rendered the best classification results producing a Cohen’s kappa score slightly larger than 0.4 as well as a total model accuracy of approximately 0.5. The second-best performance was obtained with SVM, followed by GB and RF. Furthermore, the variability over 50 permutations was lowest for DNNs, which is reflected by the lowest standard deviation. This indicates that DNN models have the highest reliability of the models across different simulations.

Modelwise performance measures are shown separately for each of the classes, i.e., diseases, in Table 2. Whereas some diseases such as PSP, svPPA, MSA-P, bvFTD, and PD reached relatively high classification performance, other classes reached middle values, i.e., healthy controls and AD, and others relatively low performance such as lvPPA, MSA-C, and nfvPPA. Of note, CBS was characterized by the lowest performance results. The order of the modelwise performance quality across the whole cohort (DNN > SVM > GB > RF) was also observed for AD and bvFTD, whereas the other classes showed a more complex picture.

**Table 2:** Metrics for model comparison

	RF	GB	SVM	DNN
Cohen’s kappa	0.325 ± 0.036	0.358 ± 0.036	0.383 ± 0.043	0.404 ± 0.03
Accuracy	0.429 ± 0.032	0.456 ± 0.032	0.472 ± 0.038	0.496 ± 0.025

Data are reported as mean ± standard deviation

*Abbreviations*: *DNN* Deep neural network, *GB* Gradient boosting, *RF* Random forest, *SVM* Support vector machine

### Importance of brain regions

The LIME method allowed us to assess the contribution of each brain region for classifying each syndrome within a model. An entire listing with the weighting of all brain regions for all models is publically available in the project repository. In the interest of greater clarity, we display the five most important brain regions for all models for three selected pathologies with well-known atrophy patterns (i.e., AD, PSP, and svPPA; see Table 3). Note that the weighting of brain regions was averaged over all patients that were classified correctly by the respective model. All models independently identified the key regions, such as the midbrain for PSP, the inferior temporal gyrus on the left side for svPPA, and the hippocampus for AD.

**Table 3:**
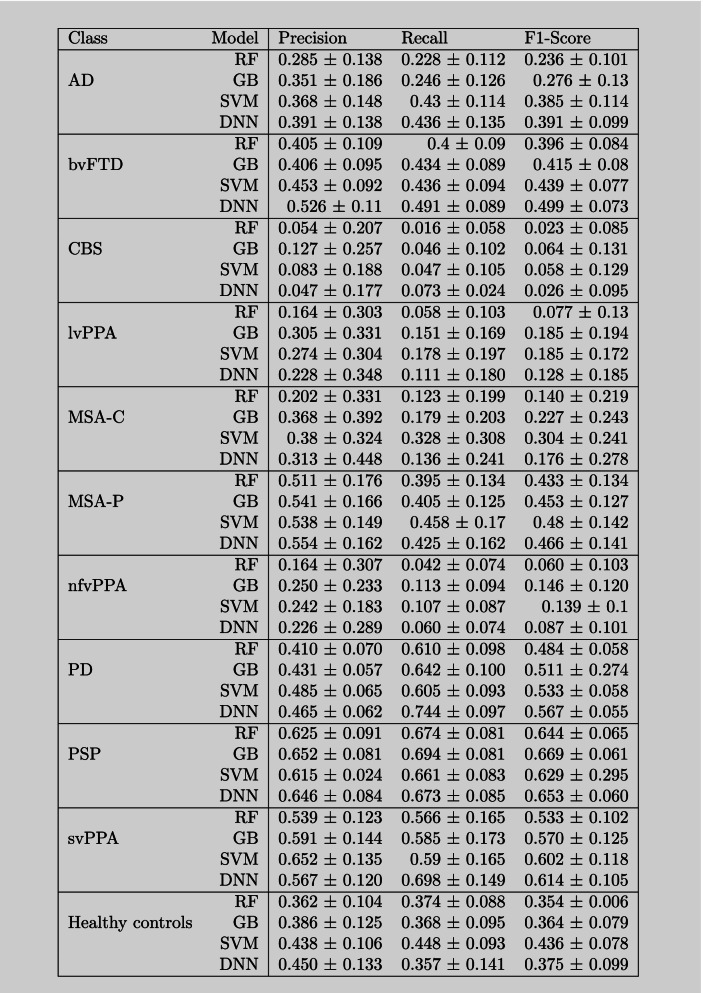
Class-wise performance metrics for multi-syndrome classification

*Abbreviations*: *AD* Alzheimer’s disease, *bvFTD* Behavioral variant frontotemporal dementia, *CBS* Corticobasal syndrome, *lvPPA* Logopenic variant primary progressive aphasia, *MSA-C* Multiple system atrophy (cerebellar dysfunction subtype), *MSA-P* Multiple system atrophy (parkinsonian subtype), *nfvPPA* Nonfluent variant primary progressive aphasia, *PD* Parkinson’s disease, *PSP* Progressive supranuclear palsy, *svPPA* Semantic variant primary progressive aphasia

## Discussion

In this work, we compared several well-established machine learning algorithms (i.e., DNN, GB, RF, and SVM) to predict the diagnosis out of numerous different neurodegenerative syndromes on the basis of pre-structured, atlas-based volumetric brain MRI data. In agreement with our hypothesis, we show that neurodegenerative diseases can be classified from structural brain imaging data, in particular, if they are characterized by specific atrophy patterns. Here, DNN showed a moderate performance, whereas the three other models showed a fair performance according to Cohen’s kappa scores. Although reasonable for this ambitious clinical question, results were not reaching substantial or even perfect classification results as achieved in comparisons of single neurodegenerative diseases vs. controls [1, 17, 27, 36–38]. This important difference between the diagnostic (disease vs. control) and differential diagnostic (disease vs. disease) approach might be related to etiological overlap between clinical syndromes, unspecific atrophy patterns for some diseases, and even the fact that single patients might show different syndromes in the course of the disease. These severe limitations, to be addressed in future studies, hamper the translation of multi-syndrome classifiers to clinical settings to date. In the following, we will discuss our results in more detail.

### Structuring imaging data for machine learning approaches

Pre-structuring of the data with atlas-based volumetry had some clear advantages such as easy assessment of particular brain regions as contributing factors for the diagnosis on an individual level as well as across syndromes, thereby increasing the interpretability of the respective model. Moreover, data could be normalized individually by adjusting to the subject’s intracranial volume. Presumably, atlas-based volumetry seems to be also superior to voxel-based morphometry, because the impact of different centers, scanner types, protocols, and applied parameters seem to be decreased by processing steps in atlas-based volumetry—a hypothesis that has to be validated in future studies. Furthermore, using volumetry data also allowed for the training of a model on a single CPU core and with 6-GB RAM. In contrast to this, the training of a convolutional neural network (CNN) with raw imaging data [39], which is the state-of-the-art method for image classification, requires machines with at least one 12-GB GPU or in case of 3D MRI volumes a server with several GPUs [40]. Finally, pre-structuring of the data increased the anonymity of the data—a general benefit that facilitates central data aggregation without risking the exposure of privacy-sensitive medical information.

The reason we were not able to conduct the same experiment with raw imaging data was that we did not have access to the raw images. Despite all the advantages of pre-structured imaging data listed above, it precludes the possibility of data augmentation of raw imaging data—a powerful strategy to increase the amount of training data and thereby boosting model performance. Furthermore, and perhaps more importantly, predefined feature extraction might lead to a loss of valuable information, which is a clear limitation of our study.

### Comparison of machine learning models

**Fig. 4:**
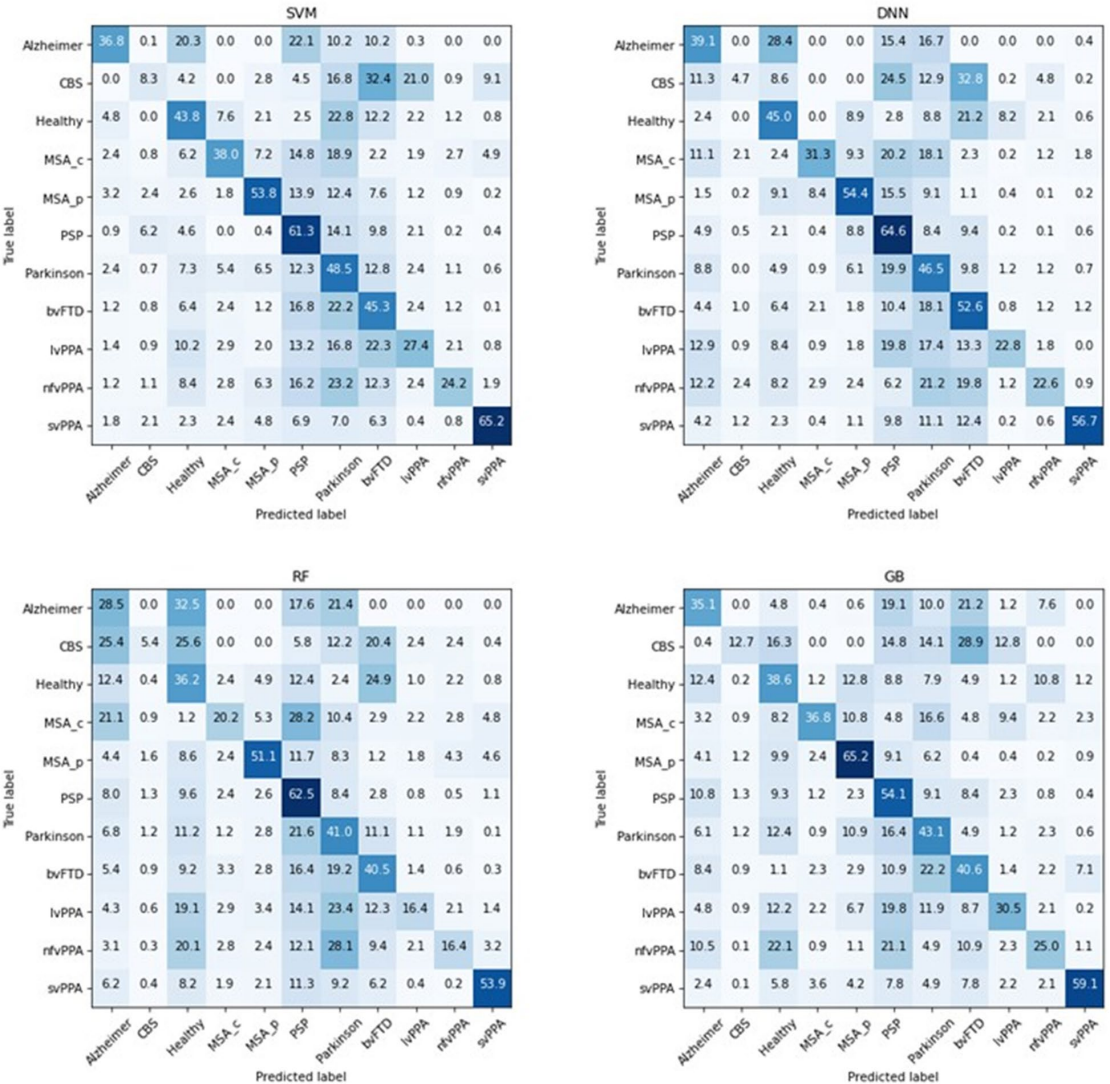
Confusion matrix for every classification model. The values are averaged over all folds and figured as a percentage value of the true label. The coloration depends on the number of predicted cases in percent (the scale goes from the highest value (dark blue) to the value 0 (white)). The confusion matrix shows row normalized percentages, which results in precision are shown for matching classes in column and row

Corresponding to the literature [41], our results indicate that the DNN with a simple feed-forward architecture is the superior method for this kind of classification task, closely followed by the SVM as illustrated in Table 2. While neural networks became the state-of-the-art method for the processing of imaging data and text data, DNNs [42] were shown to outperform tree-based methods as well as SVM with structured data. However, it is informative to take a closer look at model performance and model robustness for every single class individually, especially considering the size of the class and the specificity of atrophy patterns, respectively (see Table 3). The DNN performed best (high F1-score and high robustness) in large classes (e.g., PD, bvFTD, AD, and PSP) where there was a sufficient data for the model loss to converge. Generally, classes with smaller sample sizes expectedly led to models with weaker performance measures. GB and SVM seemed to best perform for smaller classes (e.g., MSA-C, lvPPA, CBS), while RF rendered the best robustness for smaller classes. The high robustness of RF in this case might be due to the prediction ensembles, while the superior performance of GB and SVM over the DNN might reflect those models possibly needing less data than neural networks. Notably, classes with more specific atrophy patterns (e.g., svPPA and AD) were also best predicted by the DNN despite the comparatively small sample size possible due to the faster convergence of the loss function. As expected, diseases with regionally specific and pronounced atrophy patterns such as svPPA, AD, and PSP were generally better classified than diseases with widespread and rather weak atrophy such as CBS (see Fig. 4). The confusion matrices in Fig. 4 give a nice overview of the class-specific performance of the different methods and nicely show that the DNN has a reasonable performance for all classes.

In conclusion, the larger the dataset, the better the performance. It was here, where the DNNs were able to clearly show their superiority with respect to classification performance as well as robustness. However, the point of convergence is the critical factor for good performance. For this, a balanced validation set must be used.

### Validation

The validation was not only performed by using the prediction score, but also the standard deviation of the prediction scores as a measure of robustness. Generally, the standard deviation of the model performance depends on the training dataset used, which is why we chose *k*-fold cross validation [43] instead of a leave-one-out cross-validation. In contrast to a leave-one-out cross-validation, a *k-*fold cross-validation changes the class distribution in the training dataset over the different experiments, which affects the model training. When a leave-one-out cross-validation is performed, a class imbalance in the dataset always exists in a similar ratio (with the exception of the validation instance) and is therefore reflected in a lower model quality. The highest overall robustness was observed for the DNN while the ensemble methods in turn were least robust, possibly due to their general propensity to overfit the models.

### Model performance

Both recall and precision are class-wise measures and are therewith independent of the number of true negatives, which are over-represented in a multiclass problem and thereby inflate measures contingent on the true negatives. The F1-score is a combination of both precision and recall and is supposed to give a more holistic measure of class-wise model performance.

For the overall model performance, accuracy is a popular measure, which we included in the reported metrics. However, in the case of a multiclass problem with a large imbalance, accuracy is not able to provide an honest reflection of the overall model performance. For this reason, we limit the consideration to the Cohen’s kappa score for the overall model evaluation (Table 2), because this score allows a normalization by the size of the respective class [34]. For the interpretation of the Cohen’s kappa score, the following scheme can be used: 0–0.20 as slight, 0.21–0.40 as fair, 0.41–0.60 as moderate, 0.61–0.80 as substantial, and 0.81–1 as almost perfect agreement [44]. According to this scheme, every DNN performs with a moderate performance and the three other models with a fair performance. The confusion matrix (Fig. 4) further visualizes how the DNN performs better for all different classes in comparison with the tree-based methods, where the model overfits towards the larger classes such as Parkinson, PSP, and bvFTD.

### Feature importance

To better understand the process of decision-making of every model, we extracted the feature importance with the LIME method. LIME explains the model performance by approximating an explainable model that has exactly the same predictive behavior as the used classifier.

Despite the differences in performance metrics, all methods were able to reproduce well-known atrophy patterns of respective syndromes (see Table 4). Note that unlike in binary disease-vs.-healthy classification tasks, the interpretation of the feature importance resulting from a multiclass classification problem is more ambiguous. The “important features” listed above merely reflect which brain regions were most important to differentiate the respective diagnosis from all other diagnoses included in the classification task.

**Table 4:**
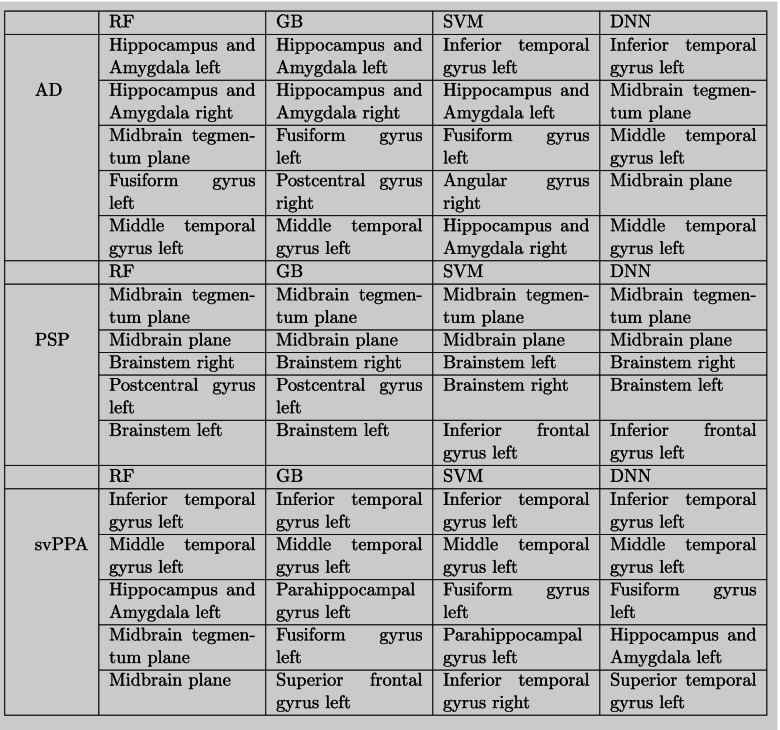
Brain regions with the highest weighting, i.e., importance, for classification

*Abbreviations*: *AD* Alzheimer’s disease, *DNN* Deep neural network, *GB* Gradient boosting, *RF* Random forest, *SVM* Support vector machine, *PSP* Progressive supranuclear palsy, *svPPA* Semantic variant primary progressive aphasia

### Limitations

While the use of volumetry data simplifies the task of classification, it simultaneously limits the classification basis to atrophy patterns only and excludes brain tissue that has no effect on atrophy. The two-stage approach consisting of the volumetry calculation and the classification of the diseases also carries the risk of error summation, which can lead to increased prediction error compared to approaches that are using the original data. Our study results might be limited by the unbalanced dataset, i.e., varying numbers in subjects per group. Although this variability reflects, at least partly, differences in prevalence and data availability, the findings of our study shall be validated in future more comprehensive, better balanced, and preferably international cohorts. Herewith, our results have to be validated externally to improve model generalization.

## Conclusion

In conclusion, we found the DNN to be the best method to assess imaging-derived structured data. However, the performance of different methods largely depends on the dataset and the underlying classification problem. To select the optimal method, one should test and validate several methods and consider the available computing resources. Despite the mentioned advantages of pre-structuring brain data, our future work will extend the application of CNN [39] on raw MRI data as well, for which remarkable results have previously been achieved for the diagnosis of smaller numbers of neurodegenerative diseases [36, 45–49]. This addresses the aforementioned limitations; however, challenges arise in data privacy when processing the data and in the increased demand of training data, which requires further data acquisition considering the rarity of coverage of the various neurodegenerative syndromes.

## Data Availability

Project repository: https://github.com/Leoniela/Comparison-ML-Algorithms-Neurodegen
